# Early Treatment of Anterior Crossbite with Eruption Guidance Appliance: A Case Report

**DOI:** 10.3390/ijerph17103587

**Published:** 2020-05-20

**Authors:** Marianna Pellegrino, Silvia Caruso, Tiziana Cantile, Gioacchino Pellegrino, Gianmaria Fabrizio Ferrazzano

**Affiliations:** 1 Unit of Dentistry, Vita-Salute San Raffaele University, 20132 Milan, Italy; 2 Department of Health, Life and Environmental Science, University of L’Aquila, 67100 L’Aquila, Italy; silvia.caruso@univaq.it (S.C.); gioacchinopellegrino61@gmail.com (G.P.); 3 Department of Neuroscience, Reproductive and Oral Sciences, School of Paediatric Dentistry, University of Naples, Federico II, 80138 Naples, Italy; tizianacantile@yahoo.it (T.C.); ferrazzano@unescochairnapoli.it (G.F.F.); 4 UNESCO Chair in Health Education and Sustainable Development: Oral Health in Paediatric Age, University of Naples Federico II, 80138 Naples, Italy

**Keywords:** eruption guidance appliance, class III malocclusion, early orthodontic treatment, EGA, anterior crossbite, interceptive orthodontics

## Abstract

The purpose of this investigation was to show how to manage an anterior crossbite in early mixed dentition with an eruption guidance appliance (EGA). The analyzed clinical case reported an anterior crossbite, a bimaxillary retrusion tendency, and a horizontal growth pattern. The anterior crossbite was an unfavorable occlusal condition that could lead to a class III malocclusion growth pattern. An early treatment approach was suggested to reach a correct sagittal jaw relationship. Hence, the selected approach acted on the dentoalveolar sector, aiming to have effects on the posterior vertical dimension and to improve the sagittal jaw’s relation. An EGA was selected to treat the patient in early mixed dentition. After 7 months of therapy with night-time use, the dental malocclusion was completely resolved. The patient continued to be treated with the same device, used as active retention. With the EGA treatment, the erupting forces, rather than the active forces, were used to resolve the dental malocclusion. This approach allowed a low compliance requirement and had a minimum psychosocial and psychological impact on the patient. The early treatment was essential to give a functional occlusion and a good balance of the soft perioral tissues and muscles.

## 1. Introduction

Dental malocclusion is a condition characterized by abnormal relationships among the teeth or dentition. A malocclusion in primary dentition is a determinant factor of malocclusion in permanent dentition [1]. The anterior crossbite is a discrepancy on the sagittal plane, which causes the forward shift of the mandible. This unphysiological position of the lower jaw can favor a class III growth pattern. Class III malocclusion generally consists of hypoplasia or retrusion of the maxilla and/or prognathic mandible, and deficient maxilla accounts for 42–63% [2]. Regardless of the growth direction of both jaws and the functional or genetic involvements, the class III relationship should be treated early [3]. It is important to minimize the aesthetic impact that the malocclusion may have on the social life of the patient, especially during adolescence. Malocclusion may lead to low self-esteem in later years, as children with primary dentition undergo a period during which they establish self-identity and early personality [4]. Furthermore, early treatment in early development is essential to avoid or to reduce the malocclusion severity and to improve therapeutic outcomes. A sagittal discrepancy of more than 7 mm, in fact, has a high risk of relapse [5]. Early intervention on the maxilla is recommended to try to reach a correct sagittal relation before the maxillary sutures close [3,6,7]. A class III malocclusion, if allowed to develop, tends to worsen with age because the growth of the mandible exceeds the growth of the maxilla. The anterior crossbite favors such a situation [6,8]. The orthodontic treatment aims to correct the sagittal, the transversal and the vertical relation between the upper and the lower jaws. Several approaches have been used to treat anterior crossbite and class III malocclusions. Some approaches adopt functional appliances, which use muscle force to correct jaw relationship [9]. Other ones use orthopedic procedures, such as Fränkel III [10], a facemask, which appears to be the most effective treatment [11], the association of splints with class III elastics and chin-cups (SEC III) [12,13], a Rapid Palatal Expander (RPE) [14] or bone-anchored maxillary protraction [15,16], which uses high force to stimulate the three-dimensional maxillary growth [17]. After 10 years of follow-up, the predictability of the orthopedic treatment, with a sagittal approach, decreases and some changes might not be maintained [2,18,19]. Vertical plane values can influence the diagnosis, the prognosis and the treatment planning of sagittal-plane-related malocclusions [20,21,22]. 

Eruption guidance appliances (EGA) with differential occlusal thickness are generally used to treat class I and class II malocclusions. Only one study in the literature reports that the anterior crossbite can be treated with these kinds of oral devices [23]. The purpose of this investigation was to show how to manage an early, unfavorable occlusal condition, such as the anterior crossbite, with an eruption guidance appliance (EGA).

## 2. Materials and Methods

### 2.1. Case Presentation

A 5-year-and-10-month-old male reported with forwardly placed lower front teeth, which worried the patient’s parents. He was in good health; his medical history showed no significant abnormalities and he had never done orthodontic treatment before. The patient’s father presented a skeletal class III malocclusion, implying a genetic predisposition. The boy remained at about the 75th percentile for height and weight over the entire period of observation.

### 2.2. Clinical Findings

**Figure 1:**
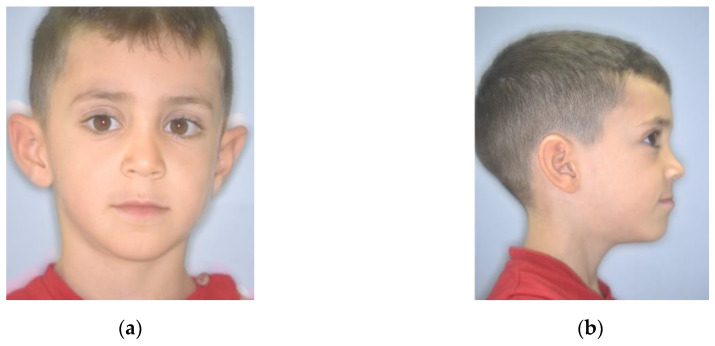
Extraoral pretreatment pictures: (**a**) frontal view; (**b**) lateral view.

On clinical extraoral examination, the patient had a straight profile, good face proportions, a flat cheekbone contour, an open nasolabial angle and a regular labiomental angle (Figure 1).

**Figure 2:**
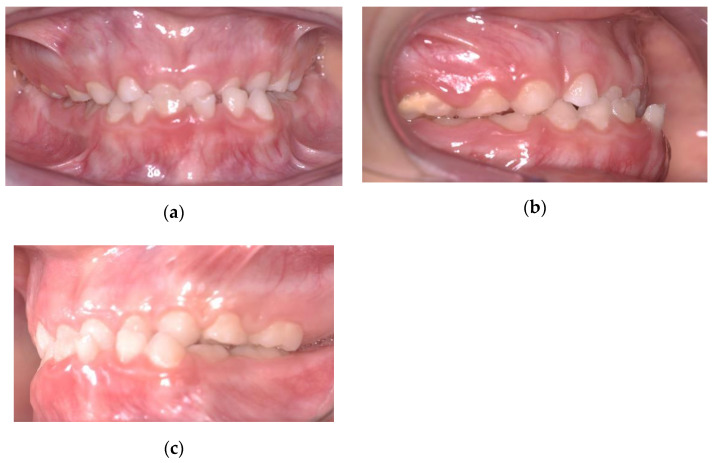
Intraoral pretreatment pictures: (**a**) frontal view; (**b**) lateral view of right side; (**c**) lateral view of left side.

He did not have clinical symptoms and the articular examination did not reveal any problems. On clinical intraoral examination, the patient was found to be in early mixed dentition. On the sagittal plane, there was a mesial step on the deciduous molars, a canine class I on the right side and a canine class III on the left side, with an anterior crossbite with 1 mm negative overjet. Around the transversal plane, he presented a 3-mm deviation of the mandibular midline towards the left, while the maxillary dental midline was coincident with the facial midline. During the opening of the mouth, the upper and lower jaw midlines became coincident and the chin deviation disappeared. This was the sign of a functional problem and not a skeletal one. Around the vertical plane, a flat Spee curve and a deep bite were noticed. Oral hygiene had to be improved (Figure 2).

### 2.3. Diagnostic Assessments

**Figure 3:**
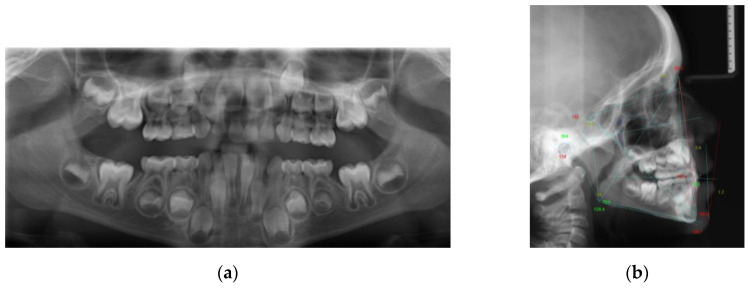
Pretreatment radiographic records: (**a**) orthopantomogram; (**b**) lateral cephalogram.

Lateral cephalogram and orthopantomogram were taken (Figure 3) and the following analyses were performed:

- MBT analysis
- Giannì analysis
- Wits appraisal
- Jarabak and Fizzel polygon.

The cephalometric analysis (Table 1) highlighted a class I with a class III tendency (Wits = −6 mm) with a biretrusion (SNA = 74°; SNB = 73°). An increased anterior vertical dimension (SN^GoGn = 38°; SnaSnp^GoGn = 32°) due to a postinclination of the mandibular plane was associated to a hypodivergent growth pattern (SAr^ArGo = 134°; ArGo^GoN = 58°). A retroinclination of upper incisors (+1^SnaSnp = 76°; +1^Occl = 93°) and an increased interincisal angle (+1^−1 = 166°) were recorded. 

### 2.4. Prognosis

The class III prognosis is hardly predictable, meaning that it is difficult to predict the severity of the problem. It depends on genetic and environmental factors. If environmental factors have a major influence, there is a greater chance to control them with orthodontic therapy and to have a better outcome [2,18,19]. Reaching a correct occlusion, with an appropriate overjet and overbite values, is fundamental to avoid dentoalveolar compensation and to favor orthognathic growth. The hypodivergent growth pattern is a positive prognostic factor.

### 2.5. Therapeutic Intervention

**Figure 4:**
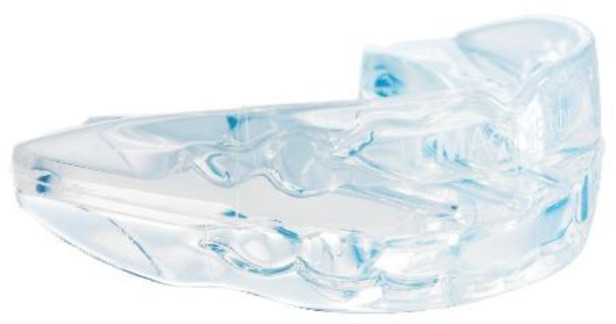
LM-Activator High Short (LM-Instruments Oy, Parainen, Finland).

The treatment goals were to resolve the anterior crossbite, reducing the class III relationship, to center the midline, to establish a stable occlusion and to improve the profile line. The treatment selected for this patient has provided for the use of a functional appliance. An EGA was chosen, specifically an LM-Activator High Short (LM-Instruments Oy, Parainen, Finland) (Figure 4).

The adopted device had a higher bilateral thickness on the molar sector, compared to the anterior one. Even though the intraoral device was preformed, the customization aspect was in choosing the appropriate model and size. Finally, a LM-Activator High Short 30 was selected. The appliance was for night-time use only; in particular, it had to be fitted soon after dinner until the next morning. During the first weeks, afternoon use also was strongly suggested, to adapt sooner to using the device. The afternoon usage was discontinued when the patient did not lose the appliance anymore during the night. The orthodontic check-up was fixed every 3 months only after verifying that the daily use was reached and correctly done. 

## 3. Results

**Figure 5:**
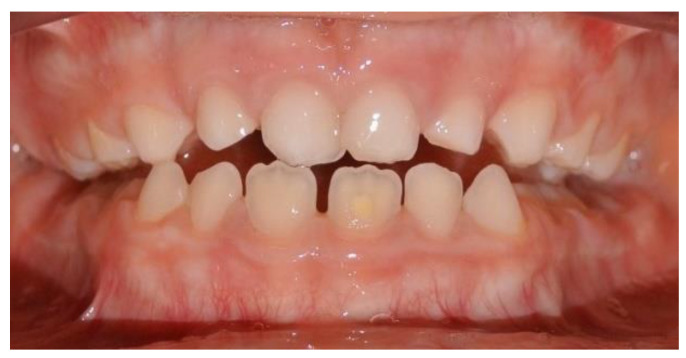
Intraoral frontal picture: edge-to-edge occlusion obtained after 4 months of treatment.

After 4 months, the patient presented a double occlusion, one in the anterior crossbite and the other one closing edge-to-edge on the frontal incisors (Figure 5).

**Figure 6:**
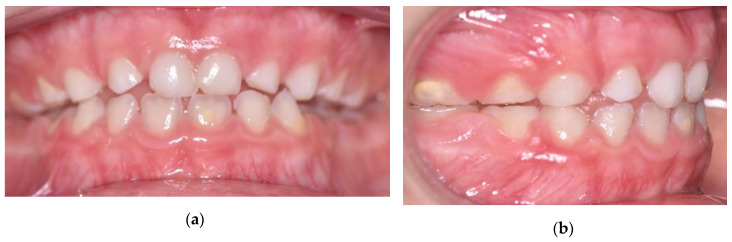

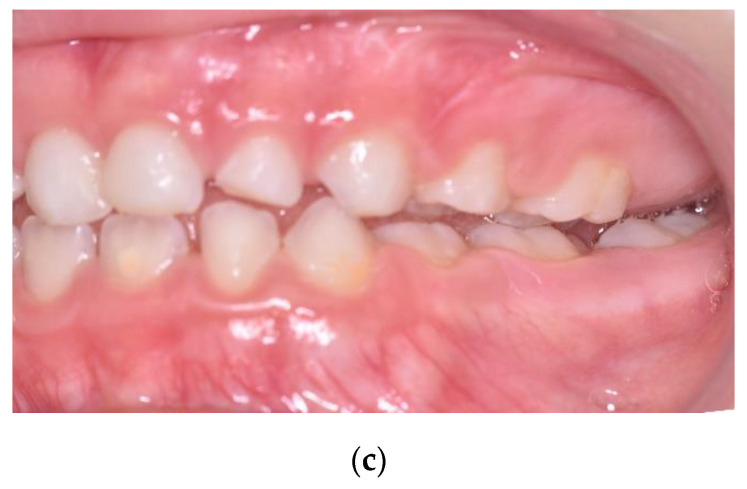
Intraoral post-treatment pictures: (**a**) frontal view; (**b**) lateral view of right side; (**c**) lateral view of left side.

This was a sign that the appliance was operating. After 7 months from the beginning of the therapy, the anterior crossbite was resolved. The initial negative overjet and deep bite were treated. The mandible’s functional deviation disappeared and so the lower midline deviated 2 mm on the right, the opposite side of the original deviation (Figure 6).

**Figure 7:**
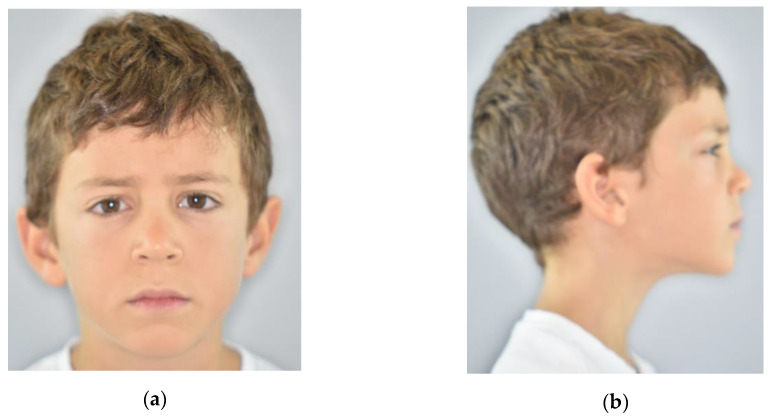
Extraoral post-treatment pictures: (**a**) frontal view; (**b**) lateral view.

The profile became harmonic (Figure 7).

### Follow-Up

The patient is continuing to use the EGA during the night as a method for retention. A bigger size was selected because of the molars’ eruption (LM-Activator High Long). This approach aims to keep an intermaxillary correct relation and to favor a three-dimensional growth of the dentoalveolar processes. The correction of the occlusal plane has to be preserved over time. 

## 4. Discussion

The functional anterior crossbite has an unfavorable effect on the growth pattern because it can lead to the development of a skeletal problem. In this condition, the mandible slows down the upper maxilla growth and its advanced position favors the mandible’s forward growth. This situation has to be interrupted as soon as possible, reaching a correct occlusal relation, to be maintained over time [24,25,26]. Furthermore, the familiarity with a class III malocclusion could genetically predispose the patient to this kind of malocclusion [27,28,29,30].

The selected early treatment aimed to reproduce an ideal sagittal condition, acting on the vertical dimension and using the eruption forces. The anterior crossbite correction could be realized because of the dental disclusion while wearing the intraoral appliance. In this way, the incisor teeth can return to their physiological position. Moreover, this EGA has some slots in the frontal area, which correct the incisal erupting trajectory. The incisal inclination change of the teeth exploits the principle of inclined planes described by Graber et al. [31]. The correct incisor position can avoid the expression of the mandibular growth pattern only on the mandibular body, distributing it also on the branch.

The occlusal plane is the most important component affecting the lower face vertical dimension [21]. The bilaterally increased thickness on the molar section, typical of the used appliance, allows the extrusion of the anterior sector and reduces the eruption of the posterior one. This controlled eruption of molars and modification of the occlusal plane is associated with a counterclockwise rotation of the mandible and a slight vertical distraction of the condyle in the glenoid cavity [32,33].

A similar therapy concept is adopted by the treatment with splints, class III elastics, and chin-cups (SEC III). The SEC III approach provides a vertical dimension control, using a thinner ramp on the anterior sector, which causes an anterior rotation of the mandible [12,13]. The Delaire facemask, instead, uses a different approach to cure skeletal class III malocclusions. It applies active orthopedic forces on the maxilla which are inclined to the occlusal plane, resulting in forces directed forwards and downward [34]. This movement trajectory does not control the molars’ eruption with a consequent clockwise rotation of the mandible. This rotation is accepted in normodivergent and in hypodivergent patients, but it is not appropriate in hyperdivergent subjects. For this reason, in hyperdivergent patients, a bite-block appliance in the mandibular arch should be associated with a facial mask to prevent mandibular rotation with progressive closure of the gonial angle [35].

The therapy with EGA allows for perfect management of early, unfavorable occlusal conditions while waiting to understand if orthopedic therapy should proceed. Furthermore, the analyzed intraoral device stimulates the perioral muscles. To keep the device in the mouth, in fact, the patient has to make an effort to keep his lips closed, activating the surrounding muscles [36,37]. This muscular activation seems to stimulate a counterclockwise rotation of the mandible [5,11]. The muscular activity is also stimulated by the proprioceptive capacity of the mouth, induced by the material properties of the soft transparent biocompatible silicone.

The use of EGA can be very useful during an early approach because it makes both traditional and digital teeth impressions unnecessary. This is an important advantage since both alginate and intraoral scans can be not tolerated in early age patients. This removable and easily washable oral device can also encourage better oral hygiene and better oral health status [38]. One of the main advantages of the proposed approach is the low psychosocial and psychological impact on the patient [39].

Early orthodontic treatment with EGA can control some aspects of the growth pattern, like an anterior crossbite, which could end in a class III malocclusion.

### Limits of the Study

The main limit of this study is the absence of a radiographic control and the impossibility of making a superimposition of traces to record the skeletal changes on the skeleton. This flaw, however, is justified by the As Low As Reasonably Achievable (ALARA) principle of radioprotection [40]. It was not possible to make a control lateral cephalogram after only 7 months of treatment in a 5-year-and-10-month-old child. The second limit is the lack of a long-term follow-up.

Following the promising results of the case report, it would be desirable to carry out a clinical study in this regard, in order to clearly assess the effectiveness of dentoskeletal effects of this kind of therapy.

## 5. Conclusions

The treatment of the anterior crossbite with an Eruption Guidance Appliance aimed to have a sagittal control acting on the vertical plane, with an occlusal approach. The chosen EGA was able to correct the occlusal plane inclination, to cause a downward stretching of the condyle, and to harmonize the profile, improving self-esteem. The short-term effect of EGA is dentoalveolar. This therapeutic choice is extremely respectful of the patient’s everyday life because of the minimum psychosocial and psychological impact. All these features make EGA another solution to treat patients with anterior crossbite in early age, during the eruption of incisors.

## Figures and Tables

**Table 1:** Cephalometric data.

Parameters	Normal Range	Recorded Values
SNA	82° ± 2°	74°
SNB	80° ± 2°	73°
ANB	2° ± 2°	1°
Wits	−1 ± 2 mm	−6 mm
SN^GoGn	32° ± 2°	38°
SnaSnp^GoGn	20° ± 5°	32°
FMA	25° ± 2°	23°
SAr^ArGo	143° ± 3°	134°
ArGo^GoN	50° ± 5°	58°
NGo^GoGn	70° ± 5°	71°
+1^SnaSnp	110° ± 3°	76°
IMPA	90° ± 2°	86°
+1^−1	130° ± 5°	166°
+1^Occl	60° ± 2°	93°
−1^Occl	70° ± 2°	72°
U1/A.Pog	5 mm ± 2 mm	−1 mm
L1/A.Pog	2 mm ± 2 mm	−5 mm
Nas^Lab	102° ± 8°	113°
SL/Sn-Pog c	−0.5 mm ± 1 mm	−3 mm
LL/Sn-Pog c	0 mm ± 1 mm	+1 mm

Sna: spina nasalis anterior; Snp: spina nasalis posterior; FMA: Frankfort-mandibular plane angle; +1: upper central incisor; -1: lower central incisor; Occl: occlusal plane; U1: upper central incisor; L1: lower central incisor; Nas: nasal angle; Lab: Labial angle; SL: superior lip; LL: lower lip; Sn: sub-nasal point; Pog c: cutaneous pogonion.
